# Cryptic diversity within two widespread diadromous freshwater fishes (Teleostei: Galaxiidae)

**DOI:** 10.1002/ece3.11201

**Published:** 2024-05-23

**Authors:** Charlotte Jense, Mark Adams, Tarmo A. Raadik, Jonathan M. Waters, David L. Morgan, Leon A. Barmuta, Scott A. Hardie, Bruce E. Deagle, Christopher P. Burridge

**Affiliations:** ^1^ Discipline of Biological Sciences, School of Natural Sciences University of Tasmania Hobart Tasmania Australia; ^2^ Evolutionary Biology Unit South Australian Museum Adelaide South Australia Australia; ^3^ School of Biological Sciences The University of Adelaide Adelaide South Australia Australia; ^4^ Department of Energy, Environment and Climate Action Arthur Rylah Institute for Environmental Research Heidelberg Victoria Australia; ^5^ Department of Zoology University of Otago Dunedin New Zealand; ^6^ Centre for Sustainable Aquatic Ecosystems, Harry Butler Institute Murdoch University Murdoch Western Australia Australia; ^7^ Australian National Fish Collection CSIRO National Research Collections Australia Hobart Tasmania Australia

**Keywords:** colonisation, delineation, *Galaxias*, gene flow, geographical barriers, imperilled, species boundaries

## Abstract

Identification of taxonomically cryptic species is essential for the effective conservation of biodiversity. Freshwater‐limited organisms tend to be genetically isolated by drainage boundaries, and thus may be expected to show substantial cryptic phylogenetic and taxonomic diversity. By comparison, populations of diadromous taxa, that migrate between freshwater and marine environments, are expected to show less genetic differentiation. Here we test for cryptic diversity in Australasian populations (both diadromous and non‐diadromous) of two widespread Southern Hemisphere fish species, *Galaxias brevipinnis* and *Galaxias maculatus*. Both mtDNA and nuclear markers reveal putative cryptic species within these taxa. The substantial diversity detected within *G. brevipinnis* may be explained by its strong climbing ability which allows it to form isolated inland populations. In island populations, *G. brevipinnis* similarly show deeper genetic divergence than those of *G. maculatus*, which may be explained by the greater abundance of *G. maculatus* larvae in the sea allowing more ongoing dispersal. Our study highlights that even widespread, ‘high‐dispersal’ species can harbour substantial cryptic diversity and therefore warrant increased taxonomic and conservation attention.

## Graphical abstract

Our study investigates the genetic diversity of *Galaxias brevipinnis* and *Galaxias maculatus*, two Southern Hemisphere fish species with both diadromous and non‐diadromous populations. Mitochondrial DNA and nuclear markers reveal the presence of cryptic species within both taxa. Our findings demonstrate that widespread migratory species can possess significant cryptic diversity, emphasising the need for increased taxonomic and conservation attention.

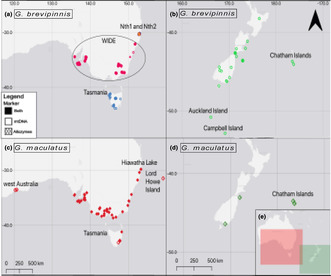

## 1 INTRODUCTION

A major impediment to the conservation of biodiversity is the presence of cryptic species, which is the occurrence of multiple species erroneously classified as a single species due to a lack of observed or quantified genetic and/or morphologic differentiation (Delić et al., 2017). Accurate species designation and identification is important for estimating the impact of threats on populations and biodiversity (Fiser et al., 2018) and the effectiveness of conservation actions (Hoekzema & Sidlauskas, 2014). Species' ranges and area of occurrence are generally overestimated when cryptic species are present, potentially diminishing their conservation status (Delić et al., 2017). Despite their often‐close evolutionary affinities, cryptic species may also have different environmental needs and provide different ecosystem services (Fiser et al., 2018). Cryptic species can contribute to the underestimation of anthropogenic extinctions, which impedes our understanding of these processes and their potential mitigation (Delić et al., 2017).

Identification of cryptic diversity has increased largely due to the application of molecular systematic approaches to both neglected and well‐studied taxa (Brown et al., 2007; Kochanova et al., 2021; Pfenninger & Schwenk, 2007; Roca et al., 2001). For example, nine lineages were found in the *Gehyra nana* species complex of geckos with overlapping distributions in several lineages across the Australian Monsoonal Tropics (Moritz et al., 2018). Widespread, polymorphic species appear particularly prone to the presence of cryptic diversity (Adams et al., 2014; Hammer et al., 2013), and many cryptic species have become morphologically diagnosable once their existence was revealed using molecular genetic or other data (Hammer et al., 2013; Raadik, 2014). However, in some instances cryptic species remain morphologically undiagnosable (Craig et al., 2009; Egge & Simons, 2006; Eitel et al., 2013; Kon et al., 2007), and some researchers question the merit of formally describing candidate species solely on molecular data (Struck et al., 2018).

Mitochondrial DNA (mtDNA) is often used to resolve taxonomic uncertainties and determine population structure in many animals because of its high rates of mutation and genetic drift (Ardren et al., 2010; Kon et al., 2007; Li et al., 2006). However, hybridisation and incomplete lineage sorting can each result in mtDNA gene trees being incongruent with their underlying species tree (Frankham et al., 2002). Surveys of nuclear genetic markers in conjunction with mtDNA are therefore desirable to provide multiple independent perspectives on taxonomic uncertainties (Grechko, 2013; Hammer et al., 2013; Wan et al., 2004). Historically, allozymes were the most widely used nuclear markers for delineating species due to the ease of screening ~30–60 independent loci (Adams et al., 2014; Hammer et al., 2007, 2013). Although now largely replaced by high‐throughput DNA sequencing of nuclear markers, allozymes have still proven insightful for delineating species and major phylogeographic breaks within species (Hammer et al., 2019; Unmack et al., 2017).

Molecular assessment of cryptic diversity is important for freshwater biodiversity. Although freshwater environments only compromise ~0.3% of the Earth's surface, they harbour disproportionally high biodiversity (e.g., almost 50% of all described fish species; Reid et al., 2013). Despite this limited extent of habitat, many freshwater fish species remain undocumented, with ~300 new species described annually (Dudgeon et al., 2006). This hidden diversity is particularly significant given that freshwater habitats are among the most imperilled in the world, with greater rates of population decline and species extinction than terrestrial and marine taxa (Reid et al., 2013). Forty percent of known freshwater fish species are on the IUCN Red List of threatened species (Reid et al., 2013), with 2041 species categorised as vulnerable, endangered or critically endangered in 2013 (Lintermans, 2013). Key threatening processes comprise habitat degradation and fragmentation, invasive species, climate change, overexploitation and pollution (Dudgeon et al., 2006; Jelks et al., 2008; Lintermans et al., 2020).

Freshwater‐limited fish lineages often experience isolation due to their habitat constraints, leading to genetic differentiation. However, cryptic diversity is also a feature of diadromous fishes—those that migrate between marine and freshwaters—despite their typically larger population sizes and higher gene flow, which might be expected to reduce diversification. Indeed, while diadromous fishes typically exhibit comparatively low population genetic structuring (Allibone & Wallis, 1993; Burridge & Waters, 2020; DeWoody & Avise, 2000; Ward et al., 1994), diadromy can also potentially facilitate long distance colonisation and subsequent founder speciation (Burridge & Waters, 2020). Notably, for some fish species diadromy can be facultative rather than obligate (Closs et al., 2003; Feutry et al., 2013; Hicks et al., 2017). Diadromous species can also harbour landlocked (freshwater limited) populations that exhibit genetic and morphological differences from their diadromous counterparts (Chapman et al., 2006; Rojo et al., 2020; Tigano & Russello, 2022). Speciation is also considered a common outcome from landlocking (Ling et al., 2001; Ovenden & White, 1990; Wallis et al., 2001). Therefore, the potential for cryptic diversity within diadromous taxa may be under‐appreciated, and thus extinction risks underestimated.

*Galaxias brevipinnis* and *Galaxias maculatus* are widely distributed diadromous fishes that exhibit morphological and life history variation throughout their range, including the presence of landlocked populations. *Galaxias brevipinnis* is found in temperate southeast Australia (including Tasmania) and throughout New Zealand and its sub‐Antarctic islands. *Galaxias maculatus* occurs in South America, Australia and New Zealand as well as neighbouring islands such as the Falklands/Malvinas, Lord Howe, and the Chatham Islands. *Galaxias maculatus* occupies low elevation freshwater systems (Bice et al., 2019), while *G. brevipinnis* has great climbing abilities and adults can penetrate farther inland (100's km inland and elevations up to 1200 m) (Atlas of Living Australia website, n.d.; Jung et al., 2009; McDowall, 2003; McDowall & Suren, 1995). Both species have experienced major declines due to habitat loss and degradation and are targeted by fisheries for their ‘whitebait’ larvae (Bice et al., 2019; Raadik et al., 2019). Introduced salmonids can also dramatically reduce the abundance of *G. brevipinnis* through predation and displacement (Rowe et al., 2002). Both species may be experiencing localised extirpation in landlocked or isolated populations (Bice et al., 2019; Raadik et al., 2019). Therefore, it is critical to clarify the potential presence of cryptic diversity within these species which may warrant conservation actions.

Both *G. brevipinnis* and *G. maculatus* have been considered to harbour cryptic species (Delgado et al., 2019; Jung et al., 2009; Raadik, 2005; Raadik et al., 2019). Both species exhibit deep molecular divergence among landmasses, suggestive of cryptic diversity (Waters et al., 2000, 2010). DNA‐based phylogenies have also rejected monophyly for Australian and New Zealand *G. brevipinnis* (Campbell et al., 2022; Waters et al., 2010; Waters & Wallis, 2001a), and for all *G. maculatus* based on the placement of *G. rostratus* (Burridge et al., 2012). Genetic and morphological divergence has also been observed among landlocked and diadromous populations in both species (Campbell et al., 2022; Carrea et al., 2013; Dunn et al., 2020; King et al., 2003; McDowall & Frankenberg, 1981; Raadik, 2005; Rojo et al., 2020). However, despite these previous studies there has as of yet been limited investigation of regional genetic variation among populations within Australia and New Zealand. This contrasts against studies of non‐diadromous galaxiids, in which at least 15 cryptic species have been identified within *Galaxias olidus* s. l. from Australia and 12 cryptic species from *G. vulgaris* s. l. in New Zealand, many of which are extremely restricted in range and highly threatened (Campbell et al., 2022; Lintermans & Raadik, 2019; Raadik, 2014, 2019a, 2019b).

### 1.1 Aims

The primary aim of this study is to address the knowledge gap regarding genetic divergence among populations of *G. brevipinnis* and *G. maculatus* within Australia and New Zealand. This includes the first assessments for associated island populations including Lord Howe, Chatham, and New Zealand Subantarctic islands. Both mitochondrial DNA and nuclear allozyme markers were used for the first time to assess the presence of candidates for cryptic species. In both species, cryptic diversity may relate primarily to marine barriers (e.g., range disjunctions within landmasses and among islands). However, we also predict a greater propensity of cryptic diversity in *G. brevipinnis* due to its greater climbing ability and penetrance inland (sensu Raadik, 2005).

## 2 METHODS

### 2.1 Sample collection

**FIGURE 1:**
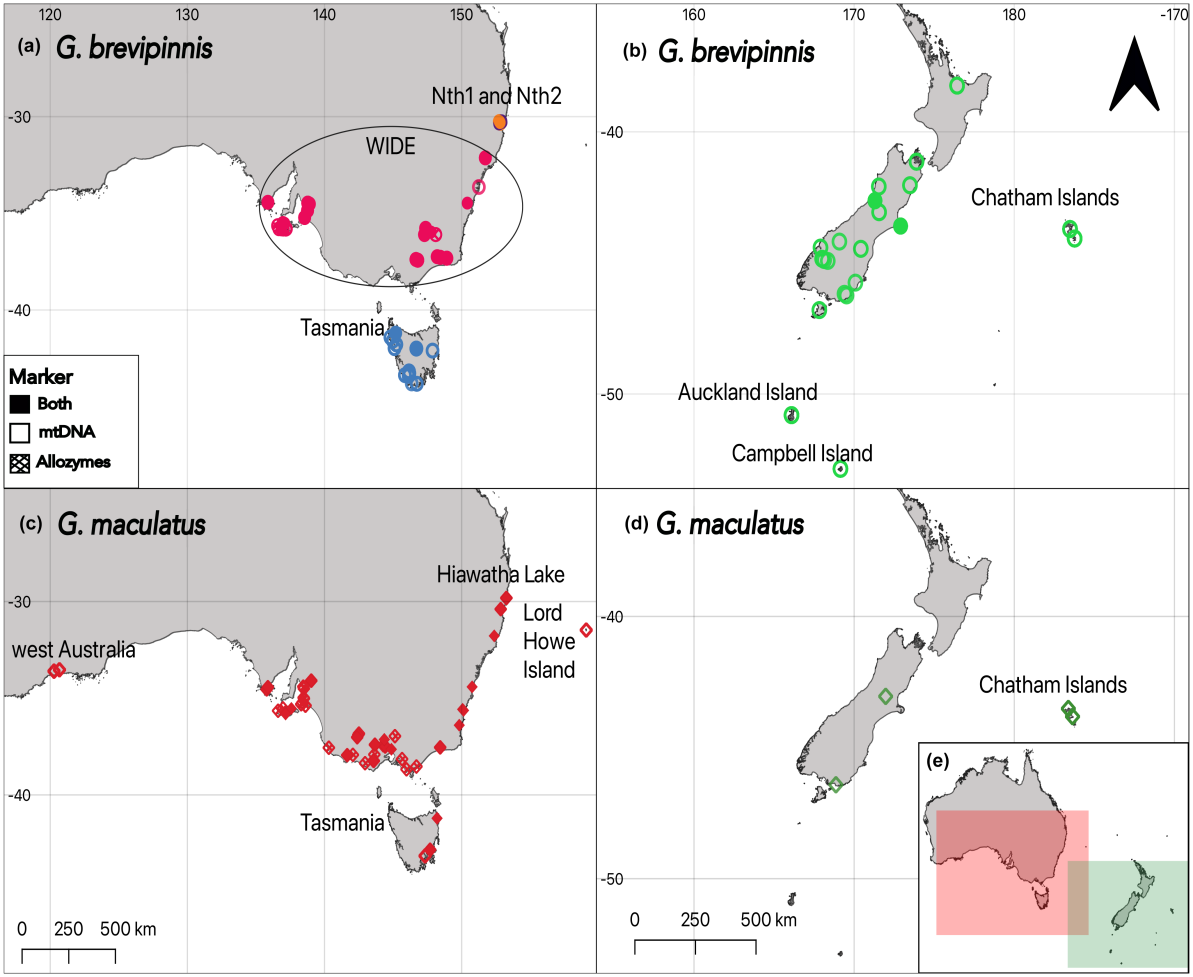
Map depicting the location of *Galaxias brevipinnis* (a, b) and *Galaxias maculatus* (c, d) samples analysed using allozymes and mitochondrial DNA (mtDNA). (a, c) sample locations in Australia; (b, d) sample locations in New Zealand. (e) Overview of locations of sample sites. Colours of the map matches other figures. A more detailed map of Nth1 and Nth2 can be found in Figure S1. See Supporting Information for more details.

A total of 259 *G. brevipinnis* individuals were utilised for allozymes (*n* = 149) and mtDNA (*n* = 149), including 14 GenBank sequences, with 39 individuals in common across data sets. The *G. maculatus* samples comprised 117 individuals: allozymes *n* = 75, mtDNA *n* = 71 (this includes 17 GenBank sequences), with 28 individuals in common across data sets. We sampled throughout their Australian ranges (52 sites for *G. brevipinnis*; 50 sites for *G. maculatus*) and included localities in New Zealand (30 sites for *G. brevipinnis*; four sites for *G. maculatus*). Among these sites were New Zealand Subantarctic Islands (Campbell Island and Auckland Island) for *G. brevipinnis*, Lord Howe Island for *G. maculatus*, and Chatham Islands for both *G. brevipinnis* and *G. maculatus* including Pitt Island for *G. brevipinnis* (see Figure 1; Tables S1 and S2). Samples were preserved in 95% ethanol or snap‐frozen using liquid nitrogen and stored at the Australian Biological Tissues Collection, based at the South Australian Museum or at the University of Tasmania.

### 2.2 Allozyme laboratory procedures

Allozyme electrophoresis was undertaken on muscle homogenates as detailed previously (Adams et al., 2014; Morgan et al., 2016; Ovenden et al., 1993). As earlier studies have shown that *G. brevipinnis* and *G. maculatus* are not close relatives, each species was screened separately. Outgroups comprised *G. olidus* (*n* = 4) for the *G. brevipinnis* study and *G. rostratus* (*n* = 3) for *G. maculatus*. The following enzymes and non‐enzymatic proteins were successfully surveyed in one or both species: ACON, ACP, ACYC, ADA, ADH, AK, ALD, AP, CA, CK, DIA, ENOL, EST, FDP, FUM, G6PD, GAPD, GDA, GLO, GOT, GP, GPI, GSR, IDH, LDH, MDH, ME, MPI, NDPK, NP, PGAM, 6PGD, PGK, PGM, PK, PEPA, PEPB, PEPD, SOD, SORDH, TPI and UGPP. Details of enzyme/locus abbreviations, enzyme commission numbers, electrophoretic conditions and stain recipes are presented in Hammer et al. (2007) and Richardson et al. (1986).

### 2.3 Analysis of allozyme data

Each allozyme data set was subjected to two types of analysis. Initially, we employed the multivariate ordination technique of principal coordinates analysis (PCoA) in a stepwise manner to identify distinct genetic lineages and instances of putative genetic admixture from first principles, that is using individuals as the unit of analysis and without a priori reference to locality or mtDNA haplotype. Full details of the philosophy and implementation of this procedure are presented in Adams et al. (2014). Thereafter, individuals were assigned to their primary genetic lineage and further grouped into sites within each primary lineage—provided there was no evidence of genetic heterogeneity within a site. Site and/or lineage‐specific quantifications were then performed: (1) the number of diagnostic differences between lineages (i.e., fixed differences, allowing a maximum tolerance of 10% for any shared alleles when summed together for a locus; see Adams et al. (2014) for the rationale underpinning this approach), and (2) Nei's unbiased genetic distance (Nei's D) between sites. A neighbour‐joining (NJ) tree was also constructed based on Nei's D. All methodological details for generating fixed difference counts, Nei's D, and NJ trees are provided in Hammer et al. (2007) and Adams et al. (2014).

### 2.4 DNA extraction and PCR

DNA was extracted using two methods: a Chelex extraction method with 200 μL of 5% Chelex and 40 μg Proteinase K where the samples were incubated at 56°C for 2 h or using the Qiagen DNeasy tissue kit according to the manufactures spin‐column protocol for animal tissue. DNA extracts were used for the amplification of the mtDNA cytochrome *b* gene using the following primers: Cytb‐Glu 5′‐GAAAAACCACCGTTGTTATTCA‐3′ and Cytb‐Thr 5′‐ CGACTTCCGGATTACAAGACT‐3′ (Waters & Wallis, 2001a). PCR was performed in 25 μL volumes containing 1x Readymix Buffer (Sigma‐Aldrich Co. LLC), 0.5 μM of each primer and 2 μL of DNA template. Thermocycling comprised 95°C for 3 min followed by 34 cycles of 95°C for 15 s, 55°C or 52°C for 15 s and 72°C for 30 s. *Galaxias maculatus* samples from west Australia were degraded, and a shorter fragment of 399 bp was amplified using primers L14724 5′‐CGAAGCTTGATATGAAAAACCATCGTTG‐3′ and H15149 5′‐AAACTGCAGCCCCTCAGAATGATATTTGTCCTCA‐3′ (Streelman et al., 2002) and 2.5 mM MgCl_2_. Thermocycling was initiated with 7 cycles of 95°C for 30s, 45°C for 30s and 72°C for 1 min prior to the 34 cycles described above but with a 48°C annealing temperature. PCR products were sent to Macrogen (Seoul, S. Korea – http://dna.macrogen.com) for purification and sequencing using Cytb‐Glu and L14724 for their specific amplicons.

### 2.5 Mitochondrial sequence analysis

Sequences were edited and aligned to a reference sequence with Geneious Prime version 2021.2 (https://www.geneious.com). Individual cytochrome *b* sequences ranged from 367 to 1145 bp, and any missing data in the alignment was coded as ‘N’. JModeltest version 2.1 was used to identify the best‐fit model of nucleotide substitution from 88 candidates using the default settings based on the corrected akaike information criterion (Burnham & Anderson, 2004). Phylogenetic analyses were performed using a Bayesian inference strategy implemented in BEAST version 2.6 (Bouckaert et al., 2019). BEAUti was used to create an input file for BEAST using the best‐fit model as determined by jModelTest 2 (Darriba et al., 2012; Guindon & Gascuel, 2003), with a relaxed‐log‐normal‐clock model and a MCMC chain length of 50,000,000 generations. Two tree priors were used to create Bayesian phylogenies: a Coalescent constant population size tree prior, suitable for intraspecific analyses, and a Yule tree prior, which assumes a constant rate of speciation and is often used for interspecific analyses. Two independent runs were conducted to test for stationarity and convergence of parameters, adequacy of burn‐in, and a sufficient effective sample size (>200) using Tracer Version 1.7 (Rambaut et al., 2018). After it was established each run converged, LogCombiner was used to combine output from the two independent runs using a burn‐in of 10%. TreeAnnotator was used to process the tree, and Figtree version 1.4 (http://tree.bio.ed.ac.uk/software/figtree/) for visualisation.

Phylogenies were also inferred using maximum likelihood in IQ‐TREE 2 (Minh et al., 2020), with 1000 bootstrap replicates and two independent runs using the best‐fit model as determined by jModelTest 2 as aforementioned. Additionally, MrBayes was run as an alternative Bayesian interference strategy because it allows for multifurcation (Huelsenbeck & Ronquist, 2001). MrBayes version 3.2.6 was run as plugin in Geneious Prime, using the substitution model most similar to those selected by jModelTest 2. For *G. brevipinnis* a MCMC chain length of 2,000,000 generations was used subsampling every 500 generations. For *G. maculatus* a MCMC chain length of 1,100,000 generations was used subsampling every 200 generations. Stationarity and convergence of parameters, adequacy of burn‐in and a sufficient effective sample size (>200) were checked in Geneious Prime. TreeAnnotator was used to process the tree using a burn‐in of 10%, and Figtree version 1.4 for visualisation.

To provide context to the geographic clustering observed in the mtDNA phylogeny, genetic distances were estimated among and within geographically concordant clades. The p‐distance has been shown to be more appropriate for quantifying genetic distances in barcoding studies than Kimura 2‐parameter distance (Srivathsan & Meier, 2012) and was quantified in MEGA X (Stecher et al., 2020) using the default settings. Because the west Australian *G. maculatus* sequences were ~400 bp, we also calculated the genetic distances with the alignment pruned to 400 bp.

As previous phylogenetic analyses have suggested that *G. brevipinnis* from Australia and New Zealand may not be monophyletic (Burridge et al., 2012; Burridge & Waters, 2020; Campbell et al., 2022; Waters et al., 2010), we included mtDNA sequences from near relatives for context, along with outgroup sequences from: *G. vulgaris* s. l. group (OQ738806–OQ738808), *G. johnstoni* (OQ738646) and *G. auratus* (JN232629.1). We also included *G. occidentalis* (OQ738863) and *G. rostratus* (JN232631.1) as outgroups for *G. maculatus* and to provide a contrast in terms of interspecific and intraspecific divergences.

## 3 RESULTS

### 3.1 Galaxias brevipinnis

#### 3.1.1 Allozyme data set

The final data set for *G. brevipinnis* comprised 149 individuals (plus four *G. olidus*) genotyped for 57 putative allozyme loci. A preliminary PCoA on all *G. brevipinnis* (Figure S2) confirmed the genetic distinctiveness of the New Zealand and Australian lineages and identified discrete ‘southern’ and ‘northern’ clusters among the Australian sites. We further explored this heterogeneity through a series of follow‐up PCoAs, each targeting various subsets of the full allozyme data set.

**FIGURE 2:**
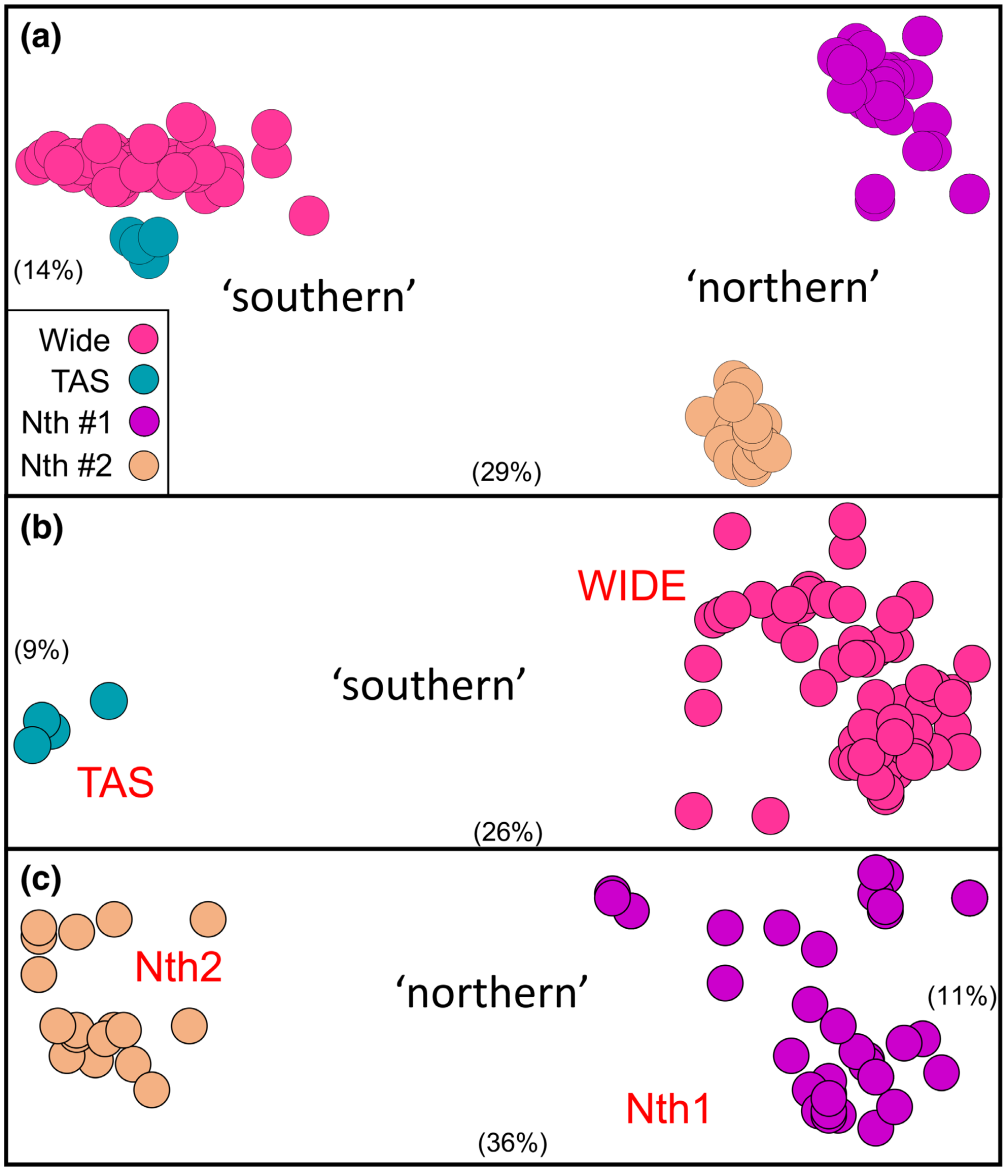
Scatterplots for the first two dimensions from the Principal Coordinates Analyses (PCoA; dimension 1 on the *x*‐axis and dimension 2 on the *y*‐axis) of the allozyme data for Australian *Galaxias brevipinnis*. Axes are scaled to reflect the relative percentage contribution of each dimension (shown in brackets). (a) initial PCoA of all 135 Australian fish; (b) PCoA of the 72 individuals comprising the ‘southern’ cluster; (c) PCoA of the 63 individuals referable to the two ‘northern’ clusters.

An initial PCoA of all 135 Australian fish (Figure 2a) revealed three primary clusters, corresponding to a ‘southern’ group for most sites plus two ‘northern’ clusters for the 12 sites representing the northern‐most catchments where *G. brevipinnis* occurs (site details in Table S1). A PCoA of the broadly distributed ‘southern’ cluster (Figure 2b) subsequently identified two distinct sub‐groups: one widespread on mainland Australia and one restricted to Tasmania, herein referred to as ‘WIDE’ and ‘TAS’, respectively. The presence of two distinctive lineages, herein referred to as ‘Nth1’ and ‘Nth2’, was also further confirmed for the ‘northern’ sites (Figure 2c). There was no evidence of genetic heterogeneity between the two NZ sites, located in the east and west of the South Island (analysis not shown). The five clusters (NZ, TAS, WIDE, Nth1, Nth2) ultimately identified by PCoA were fully diagnosable by 2–12 fixed differences (Table 1).

**TABLE 1:** Pairwise measures of diagnosability and genetic divergence among the five candidate taxa identified from the *Galaxias brevipinnis* allozyme data set with maximum sample sizes for each taxon in brackets. Lower left triangle = number of fixed differences; upper right triangle = unbiased Nei's D.

Lineage	NZ (14)	Nth1 (45)	Nth2 (18)	WIDE (68)	TAS (4)
NZ	—	0.17	0.18	0.14	0.28
Nth #1	7	—	0.07	0.07	0.20
Nth #2	7	2	—	0.08	0.20
Wide	5	4	4	—	0.14
TAS	12	9	9	4	—

The only additional substructure found via PCoA was within Nth1 (Figure S3), with individuals clustering into one of three geographically defined regions, namely (1) all sites in the Clarence River Basin, (2) all but one site in the Bellinger River Basin, and (3) the ‘Never Never’ site, close to the boundary of these two adjacent River Basins (Figure S1). The geographic structure within Nth1 was supported by 1–3 fixed differences among the three clusters identified (Figure S3). A summary of the allozyme profiles for the five main groups and the three geographic clusters within Nth1 are presented in Table S3.

Given no evidence of genetic heterogeneity within any site, the genetic affinities among individual sites were visualised using a NJ tree (Figure S4). The genetic groupings depicted are largely concordant with those identified using PCoA, with two exceptions: (1) The TAS sub‐group is nested within the Wide group, and (2) the ‘Never Never’ site is nested within the Clarence group, instead of appearing as distinct entities.

#### 3.1.2 Mitochondrial sequences

**FIGURE 3:**
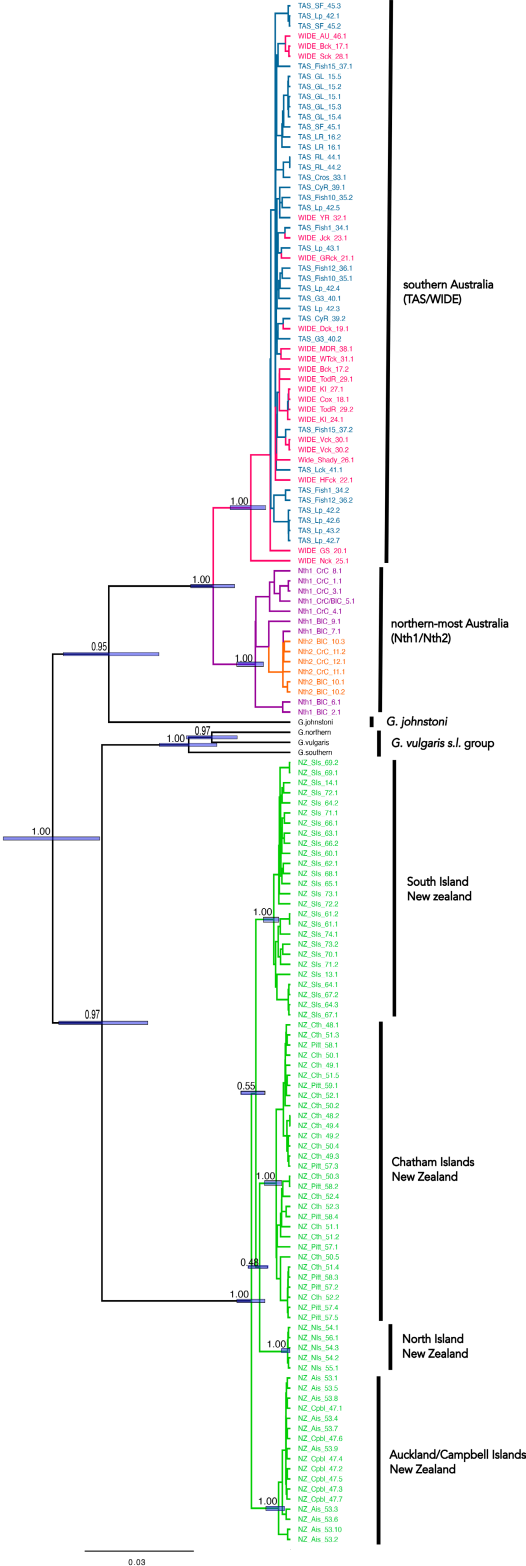
Bayesian estimate of phylogeny for *Galaxias brevipinnis* using a Coalescent prior in BEAST inferred from the cytochrome *b* region of mitochondrial DNA and rooted using *G. auratus* (not shown). Numbers represent posterior probabilities and the horizontal bars at nodes represent the 95% highest posterior density of the node height. The colours represent the candidate taxa (blue = TAS, pink = WIDE, purple = Nth1, orange = Nth2, green = NZ).

The mtDNA sequences were 618–1145 bp with an alignment length of 1145 bp across 149 sequences from *G. brevipinnis*, three from the *G. vulgaris s. l*. group, and single sequences from *G. auratus* and *G. johnstoni*. jModelTest 2 selected Tamura‐Nei+ Γ as the optimal model of sequence evolution. Three geographically concordant clades were evident from all four mtDNA phylogenies, described here following previously defined allozyme descriptions: (i) New Zealand, (ii) southern Australia (WIDE and TAS together) and (iii) northern‐most Australia (Nth1 and Nth2 together). The New Zealand and northern‐most Australian clades were each supported with posterior probabilities >0.95 in the Bayesian phylogenies and with bootstrap values of ≥70% in the maximum likelihood phylogeny (Figure 3 and Figures S5–S7). While the southern Australian clade was supported with a posterior probability of 1.00 in the Coalescent Bayesian phylogeny and a bootstrap support of 88% in the maximum likelihood phylogeny, it was unsupported in the other phylogenies. The phylogenies do not recover all *G. brevipinnis* as monophyletic, as New Zealand *G. brevipinnis* are sister to other New Zealand *Galaxias* with strong support in all but the Yule tree prior phylogeny. Further structuring was observed within New Zealand, with one clade representing both Auckland and Campbell Islands, and three separate clades representing South Island, North Island and Chatham Islands (including Pitt Island). These clades were supported across all phylogenies with exception of the Chathams clade for MrBayes and the Yule tree prior phylogeny. Relationships among these four New Zealand clades varied across phylogenies and mostly received low support. Across all of *G. brevipinnis*, the minimum between divergence between clades mentioned above was 1.33% between southern Australia and northern‐most Australia, while the maximum within‐clade divergence was 2.51% for northern‐most Australia (Table 2).

**TABLE 2:** Range of cytochrome *b* DNA sequence p‐distance for *Galaxias brevipinnis* and near relatives among (lower triangle) and within (diagonal) clades.

	*G. auratus*	New Zealand	Southern Australia	Northern‐most Australia	*G. vulgaris* group
*G. auratus*					
New Zealand	0.152–0.163	0.000–0.021			
Southern Australia	0.154–0.172	0.057–0.079	0.000–0.019		
Northern‐most Australia	0.166–0.171	0.061–0.084	0.013–0.043	0.000–0.025	
*G. vulgaris* group	0.162–0.167	0.057–0.079	0.070–0.090	0.080–0.095	0.041–0.047
*G. johnstoni*	0.160	0.070–0.086	0.054–0.072	0.061–0.073	0.083–0.088

### 3.2 Galaxias maculatus

#### 3.2.1 Allozyme data set

**FIGURE 4:**
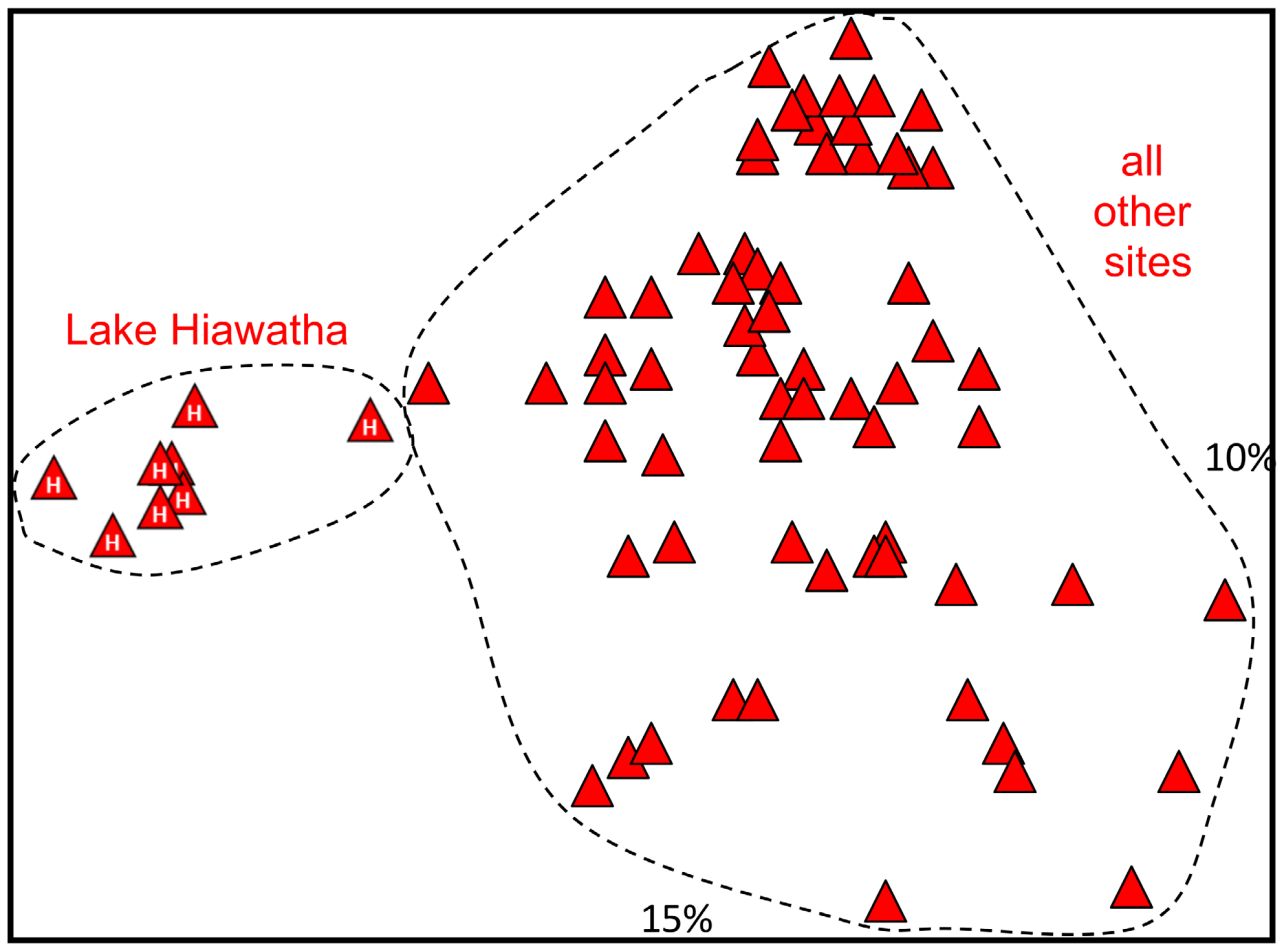
First two dimensions for the initial Principal Coordinates Analysis of allozyme variation from 75 *Galaxias maculatus*. The relative contribution of each dimension is shown alongside each axis. Base symbol as for Figure 1; individuals from the Lake Hiawatha site are overlain with the letter ‘H’.

The final allozyme data set for *G. maculatus* comprised 52 putative loci for 75 individuals from across eastern Australia plus three *G. rostratus*. As the raw data clearly demonstrated that these two sister species were unequivocally diagnosable at numerous loci (eight fixed differences; Table S4), the initial PCoA (Figure 4) was restricted to *G. maculatus*. Two marginally distinctive clusters were evident within *G. maculatus*, corresponding to (1) the Lake Hiawatha site in New South Wales, Australia, and (2) all other sites. PCoA on the latter cluster did not reveal any further genetic discontinuities. The two groups were distinguished by a single fixed difference at *Gp* and major differences in allele frequency (Δp > 40%) at *Acon*, *Got2* and *Pgm2* (Table S4). However, the NJ tree did not distinguish these two groups (Figure S8).

#### 3.2.2 Mitochondrial sequences

**FIGURE 5:**
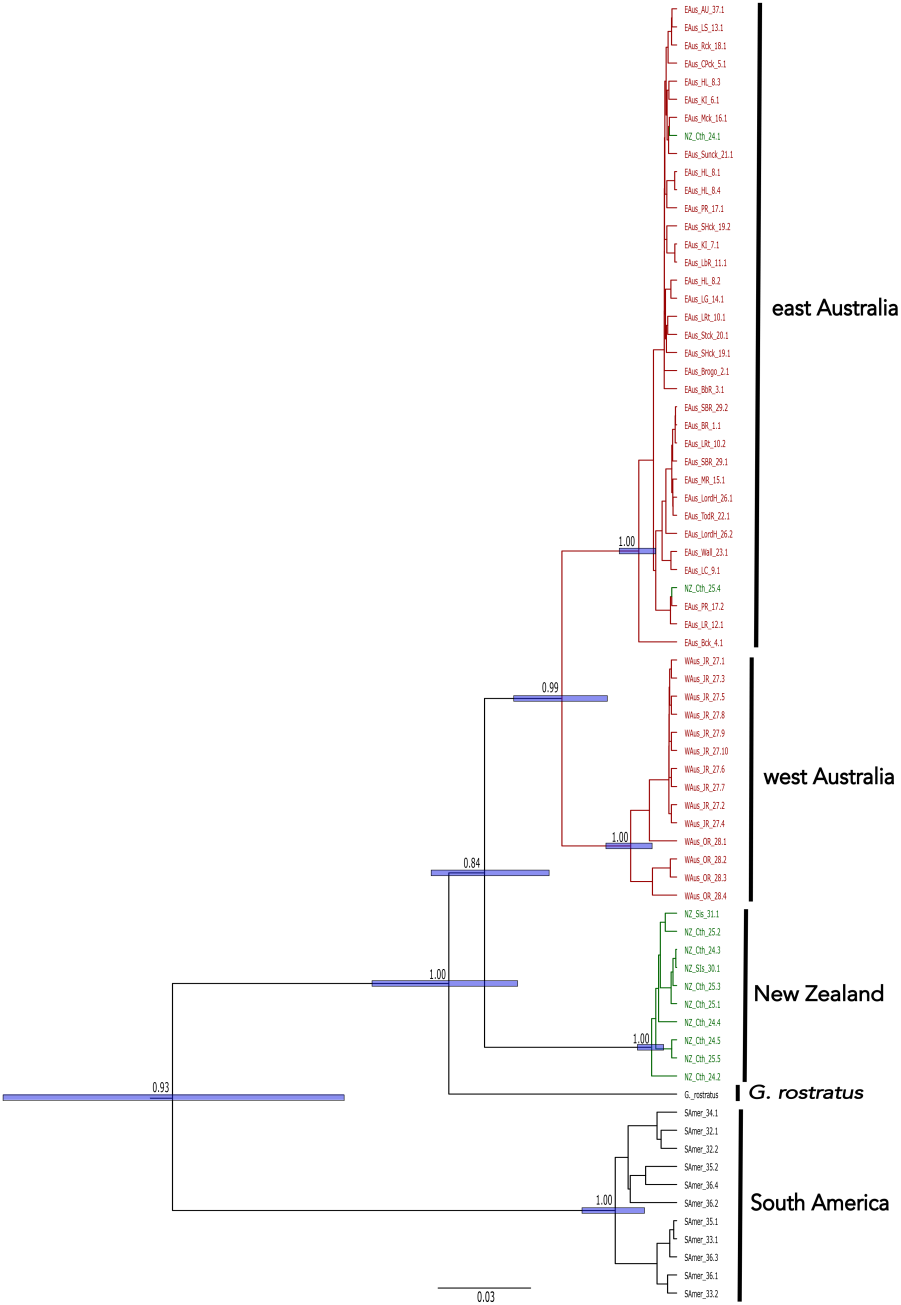
Bayesian estimate of phylogeny for *Galaxias maculatus* using a Coalescent prior in BEAST inferred from cytochrome *b* region of mitochondrial DNA, rooted using *G. occidentalis* (not shown). Numbers represent posterior probabilities and the horizontal bars at nodes represent the 95% Highest Posterior Density of the node height. Colours represent the candidate taxa (red = Australia, green = New Zealand).

The mtDNA sequences were 367–1141 bp with an alignment of 1141 bp and comprising 71 *G. maculatus* sequences from across Australia (including west Australia), along with sequences from New Zealand, and South America, plus outgroup sequences from *G. rostratus* and *G. occidentalis*. jModelTest 2 selected the best‐fit model to be Tamura‐Nei + I + Γ. Three mostly geographically concordant clades were evident from all four phylogenies: South America, New Zealand, and Australia (Figure 5 and Figures S9–S11). Each of these groups received support except for the Australian clade in the Yule tree prior phylogeny (0.92 posterior probability). In all four phylogenies, two sequences from the Chatham Islands (New Zealand) and the two from Lord Howe Island clustered closest to eastern Australian sequences, representing the disruption to geographic concordance. All phylogenies did not recover *G. maculatus* as monophyletic given the placement of *G. rostratus* as sister to Australian and New Zealand, although topological support for this was low from the Yule tree prior phylogeny. Each of the phylogenies recognised west Australia as monophyletic with topological support. However, the placement of west Australia as sister to all east Australian individuals was only recovered and supported by the coalescent Bayesian phylogeny (clustering within east Australia cannot be refuted). The minimum divergence between the east and west Australian sequences was 2.56% (2.75% based on 400 bp) while the maximum within divergence was 3.73% (3.85% based on 400 bp) for South America (Table 3). In contrast to allozymes, individuals from Lake Hiawatha did not form their own cluster relative to other east Australia sequences.

**TABLE 3:** Cytochrome b DNA sequence divergence estimates (p‐distance) for *Galaxias maculatus* and near relatives among identified (lower triangle) and within (diagonal) clades. Divergence estimates for the pruned alignment are shown in parentheses. The two Chatham islands individuals that clustered with Australia were not included here, because it notably changed the divergence estimates.

	*G. occidentalis*	South America	New Zealand	East Australia	West Australia
*G. occidentalis*					
South America	0.165–0.192	0.003–0.037			
	(0.167–0.185)	(0.000–0.038)			
New Zealand	0.179–0.201	0.112–0.154	0.000–0.015		
	(0.170–0.176)	(0.121–0.143)	(0.000–0.013)		
East Australia	0.162–0.197	0.122–0.172	0.047–0.103	0.000–0.026	
	(0.158–0.178)	(0.129–0.172)	(0.049–0.070)	(0.000–0.026)	
West Australia	0.158–0.176	0.127–0.157	0.036–0.093	0.026–0.066	0.000–0.030
	(0.156–0.173)	(0.132–0.157)	(0.038–0.093)	(0.027–0.068)	(0.000–0.030)
*G. rostratus*	0.199 (0.178)	0.125–0.148	0.070–0.097	0.077–0.094	0.085–0.093
		(0.133–0.143)	(0.074–0.083)	(0.078–0.083)	(0.088–0.093)

## 4 DISCUSSION

Freshwater‐limited fishes have become well known for their cryptic diversity (Adams et al., 2013, 2014; Hammer et al., 2014; Jerry, 2008; Kirchner et al., 2021). However, such hidden diversity is less expected for diadromous fishes given their greater dispersal abilities. Nevertheless, a few studies have revealed cryptic diversity in diadromous species (e.g., Galván‐Quesada et al., 2016; McMahan et al., 2013, 2021). Here, based on fixed differences in allozymes and relationships and divergences estimated from mtDNA sequences, we have identified cryptic diversity within two widespread and well‐studied diadromous fishes and suggest candidates for distinct taxa.

For both allozymes and mtDNA cryptic diversity is suggested within Australian *G. brevipinnis*, with two groups delineated: one lineage in southern Australia (TAS and WIDE) and one in the north (Nth1 and Nth2). Allozymes further delineate the two northern‐most groups. It is not known if the two northern candidates (Nth1 and Nth2) are diadromous, nevertheless their catchments have significant marine separation (>300 km) from other catchments harbouring *G. brevipinnis* (Raadik, 2005). Both candidate taxa also display distinctive morphology from each other and from their southern counterparts (Raadik, unpublished). Species distribution models based on the location of other *G. brevipinnis* populations and including 10 environmental factors (e.g., average rainfall, temperature, slope and elevation), failed to predict the occurrence of these northern‐most populations (Growns & West, 2008). This suggest the northern‐most populations could be locally adapted ecotypes, however more extensive sampling would be needed to fully resolve the taxonomy and reject phenotypic plasticity. The northern‐most candidate taxa also occupy the same catchment and may have partitioned the available habitat at the scale of stream reaches. Similar geographic patterns have been observed between New Zealand *G*. ‘southern’ and *G. gollumoides*, with apparent partitioning of habitat at a fine scale in the river network they inhabit (Crow et al., 2010).

The Tasmania (TAS) group is also somewhat distinctive from the other southern mainland populations (WIDE), with four fixed differences and their isolation by a marine distance of ~250 km. However, it is nested within these WIDE populations in the allozyme tree and lacks support from mtDNA. The recognition of the TAS group has been made based on previous mtDNA evidence (Waters & Wallis, 2001a). *Galaxias coxii* (Macleay 1880) and *G. weedoni* (Johnston 1883) have precedence for mainland Australia and Tasmania, respectively, but were synonymised with *G. brevipinnis* by McDowall and Frankenberg (1981). It would be beneficial for future molecular and morphological analyses to employ increased sampling of locations and loci to further assess the distinctiveness of the Tasmanian population (TAS group).

In comparison with allozymes, our mtDNA analyses were unable to distinguish the two northern‐most (Nth1 and Nth2) Australian candidate taxa from each other. This outcome most likely reflects the mtDNA gene tree/species tree discordance that is often observed among closely related species (e.g., Hammer et al., 2014; Unmack et al., 2019). Furthermore, minimum between‐clade mtDNA distances were not appreciably larger than maximum within‐clade distances (e.g., 1.33% among Australian clades versus 1.90% within the southern Australian clade). Likewise, in the case of the *G. olidus* complex, 15 species were suggested based on fixed allozyme plus morphological differences, but only eight of these were monophyletic for mtDNA, and two did not receive any topological support (Adams et al., 2014). Underestimation of species diversity from mtDNA relative to allozymes could reflect introgressive hybridisation and mtDNA capture (Moore, 1995) or incomplete lineage sorting (Avise et al., 1986). Regardless, the level of among clade cytochrome *b* divergence in *G. brevipinnis* is comparable with other cryptic species complexes (Bronaugh et al., 2020; Hoekzema & Sidlauskas, 2014; Jirsova et al., 2019), including several freshwater‐limited galaxiid radiations (Adams et al., 2014; Chakona et al., 2013; Vanhaecke et al., 2012; Waters & Wallis, 2001b; Wishart et al., 2006).

With respect to New Zealand *G. brevipinnis*, our results support previous suggestions that *G. brevipinnis* contains cryptic diversity. Additionally, our observed mtDNA relationships among New Zealand and nearby island *G. brevipinnis* populations also raise the potential significance of marine barriers for cryptic diversity in this lineage. Four reciprocally monophyletic clades are evident: (i) Auckland and Campbell Island, (ii) Chatham Islands, (iii) South Island New Zealand and (iv) North Island New Zealand. That not all clades were supported can be explained by the shallow divergence observed among them and does not preclude complete but recent genetic isolation. While our study lacked allozyme data for all these localities, we predict fixed differences given observations of their greater resolving power elsewhere in this (described above) and other studies (Adams et al., 2014; Hammer et al., 2014). Furthermore, the divergence among the subantarctic islands, Chatham Islands and the South Island New Zealand are supported by nuclear SNP evidence (Darestani et al., 2023). However, non‐diadromous South Island Lake populations also exhibit nuclear SNP distinction from each other and diadromous populations (Darestani et al., 2023), which raises the possibility that divergences observed during that study may reflect intraspecific spatial population genetic structure, rather than support for cryptic species. Nevertheless, we recommend further genetic assessments (including more vigorous sampling of the North Island) to assess the taxonomic distinction of these lineages.

Our analyses of *G. maculatus* also revealed candidates for cryptic diversity. We confirm the large genetic divergences previously observed among South American, New Zealand and Australian *G. maculatus* (Pavuk, 1997; Waters et al., 2000; Waters & Burridge, 1999), for which different subspecies have been suggested (Stokell, 1966). We also detected large divergence (2.6%–6.6%) between east and west Australia, spatially separated by ~1500 km. This genetic divergence contrasts with genetic homogeneity within east Australian *G. maculatus* at comparable spatial scales (see also O'Dwyer et al., 2021), with only some subtle divergence evident in the landlocked Hiawatha Lake population. Although we lacked allozyme data for south‐west Australian *G. maculatus*, Pavuk (1997) observed that allozyme distinction of this population exceeded that between east Australia and New Zealand, albeit based only a handful of allozyme loci, none of which displayed fixed differences. A follow‐up assessment of the taxonomic distinctiveness of south‐west Australian *G. maculatus* based on additional nuclear loci is clearly desirable, despite not being flagged to harbour cryptic diversity during conventional taxonomic treatment (McDowall & Frankenberg, 1981).

The divergence of the south‐west Australian *G. maculatus* population may reflect marine isolation, here representing the complete absence of rivers in the intervening Eucla basin, spanning ~1500 km (Unmack, 2001; Unmack et al., 2012). This region has also been implicated for divergences of terrestrial animals and plants (Guay et al., 2010; Schmidt et al., 2014), although other freshwater taxa appear to have surmounted it (Unmack et al., 2011). Alternatively, the reputedly landlocked lifecycle of south‐west Australian *G. maculatus* (Morgan et al., 2006) may have promoted genetic divergence, similar to that suggested for south‐west Australian *Galaxias truttaceus* (Morgan et al., 2016), and other populations of *G. maculatus* in south‐eastern Australia (McDowall & Frankenberg, 1981; Pollard, 1971a, 1971b) and South America at much smaller spatial scales (down to ~2 km; McDowall & Frankenberg, 1981; Pollard, 1971a, 1971b; Rojo et al., 2020; Zattara & Premoli, 2005). However, larval gene flow from east Australia would also be impeded by the east‐flowing Leeuwin current, with ocean currents also implicated for genetic structuring in South American *G. maculatus* (González‐Wevar et al., 2015).

Across their overlapping Australian range, we suggest three putative cryptic candidate taxa within *G. brevipinnis* (southern Australia (TAS and WIDE together), Nth1, Nth2) and highlight large genetic divergence within the *G. maculatus* group. This is consistent with our expectations based on the greater climbing ability of *G. brevipinnis*, allowing it to penetrate farther inland and form isolated populations. In contrast, *G. maculatus* are unable to overcome 3 m sloped passages or other inland barriers that are readily surmountable by *G. brevipinnis* (Doehring et al., 2012). This difference in number of candidate cryptic taxa also mirrors differences in the diversity of their close relatives. *Galaxias brevipinnis* has 12 close relatives in New Zealand, represented by *G. vulgaris s.l*. lineages (Campbell et al., 2022), plus *G. johnstoni* and *G. pedderensis* in Tasmania (Burridge et al., 2012). In contrast, over a much broader spatial scale on mainland Australia, the only close relatives of *G. maculatus* are *G. occidentalis* and *G. rostratus* (Burridge et al., 2012). These observations match expectations if the ancestors of the ‘maculatus’ and ‘brevipinnis’ groups had dispersal abilities similar to *G. maculatus* and *G. brevipinnis* today, respectively.

Differences in marine dispersal ability could also explain the different levels of cryptic diversity within *G. brevipinnis* and *G. maculatus*. While both species have similar lifecycles, with juveniles from diadromous populations spending 4–6 months at sea (Jung et al., 2009), *G. maculatus* populations on oceanic islands (Lord Howe, Chatham islands) provided genetic evidence for dispersal across large marine barriers, as shown by the two individuals from both Chatham and Lord Howe that cluster within Australian samples. Waters et al. (2000) also found *G. maculatus* individuals from New Zealand with closer affiliation to Tasmania. In contrast, monophyly was observed for *G. brevipinnis* in similar settings, such as for Tasmania, Chatham Islands and New Zealand sub‐Antarctic islands (Auckland and Campbell Island). Otolith signatures also suggest that the majority of diadromous *G. brevipinnis* in New Zealand recruit to their natal stream, with larvae and juveniles potentially orientating into nearshore river plumes to limit dispersal (Augspurger et al., 2021). In more insular settings, *G. brevipinnis* that stray may have limited probability of recruiting elsewhere. Additionally, even if they do recruit elsewhere, they may not leave long‐term genetic signatures (Waters et al., 2013). Furthermore, *G. maculatus* appear to have greater larval abundance at sea as they dominate the whitebait (larvae) fishery in New Zealand (McDowall, 1965; Yungnickel et al., 2020), and this confers greater gene flow.

## 5 CONCLUSIONS AND TAXONOMIC RECOMMENDATIONS

This study demonstrates additional cryptic diversity within two widespread diadromous fishes of the Southern Hemisphere. Based on these findings, we suggest the presence of several putative undescribed taxa. We recommend future steps follow the integrative species delineation framework proposed by Unmack et al. (2021) and include morphological analyses such as those described by Raadik (2014). However, it should be noted that a lack of morphological distinction does not necessarily preclude the presence of multiple species, nor the benefits of their recognition during investigations of evolutionary history and ecology (Delić et al., 2017). The differentiation we observed within *G. brevipinnis* and *G. maculatus* could be a result of (geographic) isolation, habitat complexity or ecological and life history differences such as dispersal abilities and recruitment. Assessing the migratory status of the two northern‐most candidate taxa of *G. brevipinnis*, for example is essential to understand potential drivers of diversification. These two northern‐most candidate taxa of *G. brevipinnis* require more detailed study (morphological and genetic) but based on current data, *G. brevipinnis* in that region should be recognised as a conservation unit separate from the other Australian populations as a precautionary measure. While we do not currently suggest south‐west Australia *G. maculatus* as a candidate taxon, the large genetic divergence warrants further genetic assessment and supports its recognition as a separate conservation unit. The recognition of such conservation units will help maintain potentially important genetic diversity. While this study covers a broad geographic range, finer geographic coverage may uncover other regionally distinct lineages. Indeed, with diadromous fishes particularly vulnerable to habitat loss and degradation in both freshwater and marine environments, it is essential that such cryptic diversity be identified and conserved (Jung et al., 2009).

Cryptic diversity has been previously suggested in widespread and vagile taxa—those that are less affected by barriers. This includes both non‐migratory and migratory bird species (Irwin et al., 2011; Lohman et al., 2010), planktonic marine copepods (Halbert et al., 2013), and marine and freshwater bony and cartilaginous fishes (D'Aloia et al., 2017; Fahmi et al., 2021; Neilson & Stepien, 2009). For example, migratory populations of the Wilson's warbler (a bird) exhibit strong genetic differentiation, perhaps reflecting differences in migratory patterns (Irwin et al., 2011). Similar to diadromous fishes harbouring landlocked populations as a result of the loss of their marine migratory phase, other taxa, such as birds, experience loss of migration resulting in resident populations that may live in sympatry but are reproductively isolated (Gómez‐Bahamón et al., 2020). With environmental change, loss or changes in migration pathways across taxa are to be expected and could promote diversification (de Zoeten & Pulido, 2020). Our results highlight that widespread and vagile species should be assessed to avoid erroneous recognition of species boundaries, the underestimation of endemism (Lohman et al., 2010), and inappropriate management and conservation priorities. Such assessments may correct previous over‐estimation of species abundance and range. Genetic studies such as ours can depict population structure and identify populations or conservation units with novel genetic diversity to maintain and highlight where ecological work and management efforts should be focussed.

## AUTHOR CONTRIBUTIONS

**Charlotte Jense:** Conceptualization (equal); data curation (equal); formal analysis (lead); visualization (lead); writing – original draft (lead); writing – review and editing (lead). **Mark Adams:** Conceptualization (equal); data curation (equal); formal analysis (lead); visualization (lead); writing – original draft (equal); writing – review and editing (equal). **Tarmo A. Raadik:** Conceptualization (equal); data curation (equal); writing – original draft (equal); writing – review and editing (equal). **Jonathan M. Waters:** Data curation (equal); writing – original draft (equal); writing – review and editing (equal). **David L. Morgan:** Data curation (equal); writing – original draft (equal); writing – review and editing (equal). **Leon A. Barmuta:** Writing – original draft (equal); writing – review and editing (equal). **Scott A. Hardie:** Data curation (equal); writing – original draft (equal); writing – review and editing (equal). **Bruce E. Deagle:** Conceptualization (equal); data curation (equal); writing – original draft (equal); writing – review and editing (equal). **Christopher P. Burridge:** Conceptualization (equal); data curation (equal); writing – original draft (equal); writing – review and editing (equal).

## CONFLICT OF INTEREST STATEMENT

The authors declare that they have no conflict of interest.

## Supporting information


Figures S1–S11.

Tables S1–S4.

## Data Availability

The mtDNA sequence data are available on GenBank under accession numbers OQ738671 – OQ738862.
