# Supplementary material for: Cryptic diversity within two widespread diadromous freshwater fishes (Teleostei: Galaxiidae)
Source: Ecol Evol. 2024 May 23;14(5):e11201. doi: 10.1002/ece3.11201 (PMC11116845; doi:10.1002/ece3.11201)
Supplement: Supplementary file 1 — Figures S1–S11. [file ECE3-14-e11201-s002.docx]

Supplementary Figure 1. The distribution of ‘northern’ Australian *Galaxias brevipinnis* sample sites included in the allozyme study. Lineage symbols match those used in Figure 2 (purple = Nth1, orange = Nth2), except that the Never Never site (lineage Nth1) is shown as a cross. The dark grey line indicates the boundary between the Clarence and Bellinger River Basins.

Supplementary Figure 2. Scatterplots for the first two PCoA dimensions (dimension 1 on the x-axis and dimension 2 on the y-axis) for all 149 *G. brevipinnis* screened in the allozyme study. Axes are scaled according to the relative percentage contribution of each dimension (shown in brackets).

Supplementary Figure 3. Scatterplot of ordination scores in the first two dimensions (dimension one on the x-axis and dimension 2 on the y-axis) for the initial Principal Coordinates Analysis on 45 *Galaxias brevipinnis* lineage Nth1 individuals. Samples are labelled according to broad geographic (drainage) groupings (4 = Bellinger River Basin; 5 = Clarence River Basin; N = Never Never site (n=6 fish, all with the same PCoA coordinates in the first two dimensions).


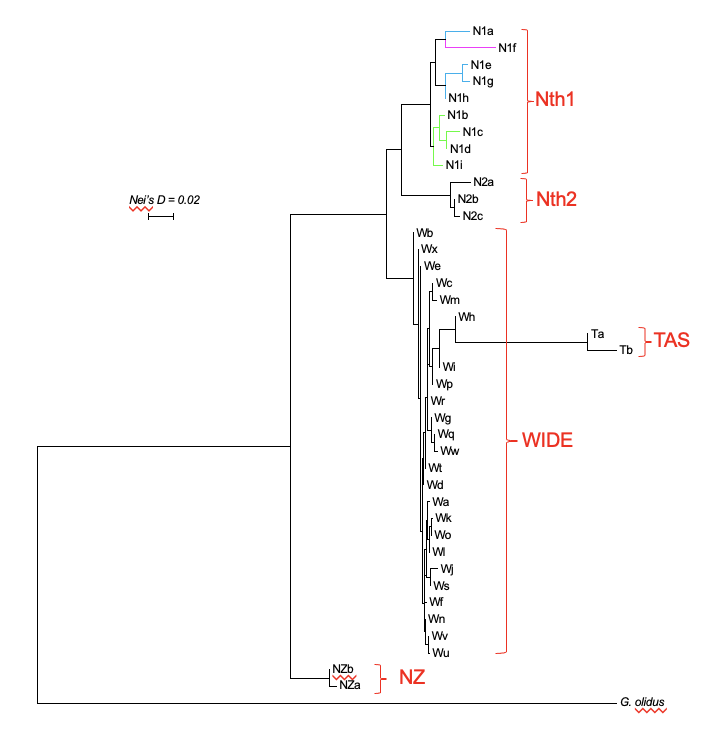


Supplementary Figure 4. Allozyme Neighbor Joining tree for *Galaxias brevipinnis*, rooted using *Galaxias olidus*. Sites are labelled according to codes given in accessory Table 1 and assigned to candidate taxa (red fonts and brackets). Sites within Nth1 are also assigned to one of three clusters identified in Supplementary Figure 1 as indicated by the colour of their terminal branches (blue = Clarence, purple = Never Never, and green = Bellinger).

Supplementary Figure 5. Bayesian estimate of phylogeny for *Galaxias brevipinnis* using a Yule tree prior in BEAST inferred from the cytochrome *b* region of mitochondrial DNA and rooted using *G. auratus* (not shown). Numbers represent posterior probabilities and the horizontal bars at nodes represent the 95% Highest Posterior Density of the node height. The colours represent the candidate taxa (blue = TAS, pink = WIDE, purple = Nth1, orange = Nth2, green = NZ).

Supplementary Figure 6. Maximum likelihood estimate of phylogeny for *Galaxias brevipinnis* inferred from the cytochrome *b* region of mitochondrial DNA and rooted using *G. auratus* (not shown). Numbers represent bootstrap support. The colours represent the candidate taxa (blue = TAS, pink = WIDE, purple = Nth1, orange = Nth2, green = NZ).

Supplementary Figure 7. Bayesian estimate of phylogeny for *Galaxias brevipinnis* using MrBayes from the cytochrome *b* region of mitochondrial DNA and rooted using *G. auratus* (not shown). Numbers represent posterior probabilities and the horizontal bars at nodes represent the 95% Highest Posterior Density of the node height. The colours represent the candidate taxa (blue = TAS, pink = WIDE, purple = Nth1, orange = Nth2, green = NZ).

Supplementary Figure 8. Allozyme Neighbour Joining tree for *Galaxias maculatus*, rooted using *Galaxias rostratus*. Sites are labelled according to the site code given in accessory Table 1. The Lake Hiawatha (New South Wales) subpopulation, diagnosable by one fixed difference from all other sites, is identified by an arrow.

Supplementary Figure 9. Bayesian estimate of phylogeny for *Galaxias maculatus* using a Yule tree prior in BEAST inferred from cytochrome *b* region of mitochondrial DNA, rooted using *G. occidentalis* (not shown). Numbers represent posterior probabilities and the horizontal bars at nodes represent the 95% Highest Posterior Density of the node height. Colours represent the candidate taxa (red = Australia, green = New Zealand).

Supplementary Figure 10. Maximum likelihood estimate of phylogeny for *Galaxias maculatus* inferred from cytochrome *b* region of mitochondrial DNA, rooted using *G. occidentalis* (not shown). Numbers represent bootstrap support. Colours represent the candidate taxa (red = Australia, green = New Zealand).

Supplementary Figure 11. Bayesian estimate of phylogeny for *Galaxias maculatus* using MrBayes inferred from cytochrome *b* region of mitochondrial DNA, rooted using *G. occidentalis* (not shown). Numbers represent posterior probabilities and the horizontal bars at nodes represent the 95% Highest Posterior Density of the node height. Colours represent the candidate taxa (red = Australia, green = New Zealand).
