# Supplementary material for: Cryptic diversity within two widespread diadromous freshwater fishes (Teleostei: Galaxiidae)
Source: Ecol Evol. 2024 May 23;14(5):e11201. doi: 10.1002/ece3.11201 (PMC11116845; doi:10.1002/ece3.11201)
Supplement: Supplementary file 2 — Tables S1–S4. [file ECE3-14-e11201-s001.zip › Supplementary_Tables.docx]

Supplementary Table 1. Information on *G. brevipinnis* individuals used for allozyme and mitochondrial DNA (mtDNA) analyses. A detailed map of all sample localities can be found here: [G. brevipinnis and G. maculatus online map](https://www.google.com/maps/d/edit?mid=1H7CHXkTkDGqd4t2NV-trR7M6SuqyyeM&usp=sharing)

Supplementary Table 2. Information on *G. maculatus* individuals used for allozyme and mitochondrial DNA (mtDNA) analyses. A detailed map of all sample localities can be found here: [G. brevipinnis and G. maculatus online map](https://www.google.com/maps/d/edit?mid=1H7CHXkTkDGqd4t2NV-trR7M6SuqyyeM&usp=sharing)

Supplementary Table 3. Allozyme frequencies for all candidate species and phylogeographic groups within *G. brevipinnis*, as identified using stepwise Principal Coordinates Analysis. Candidate taxa used are shown in black font and other groups in red font; maximum sample sizes for each taxon in brackets. For polymorphic loci, the frequencies of all but the rarer/rarest alleles are expressed as percentages and shown as superscripts. Alleles not separated by a comma all shared the frequency indicated. A dash indicates no genotype was assignable at this locus. Invariant loci: *Acyc*, *Ak*, *Ald2*, *Ald3*, *Ca*, *Ck*, *Enol1*, *Fdp*, *Idh2*, *Ldh1*, *Mdh1*, *Pgam*, and *Tpi*.

| **Locus** | **NZ**  **(14)** | **WIDE**  **(68)** | **TAS**  **(4)** | **Nth2**  **(18)** | **Nth1**  **(45)** | **Nth1**  **Bellinger**  **(22)** | **Nth1**  **Clarence**  **(17)** | **Nth1**  **Never Never**  **(6)** |
| --- | --- | --- | --- | --- | --- | --- | --- | --- |
| *Acon* | b^96^,c | c^99^,b | c | c^94^,bd^3^ | c^97^,d | c^93^,d | c | c |
| *Acp* | c | c^96^,d^3^,a | c | c | c | c | c | c |
| *Ada* | l^46^,k^36^,n^10^,mi^4^ | k^93^,fg^3^,h | f^87^,e | d^67^,j | j^35^,d^33^,h^31^,b | d^61^,h^37^,b | j^91^,d | h |
| *Adh1* | f | f^85^,c^13^,ad^1^ | d^87^,b | f^86^,g | f^92^,d^6^,a | f^89^,d | f^94^,a | f |
| *Adh2* | b | b^99^,a | b | b | b | b | b | b |
| *Ald1* | b^82^,a | b | b | b | b | b | b | b |
| *Ap1* | a | a | a | a | a^99^,b | a^98^,b | a | a |
| *Ap2* | b | b^94^,a | b | b | b | b | b | b |
| *Dia* | c^96^,b | b^55^,c^25^,d^17^,a | d^75^,b | b^84^,a^9^,c | b^71^,c^16^,a^9^,d | b^53^,c^39^,d | b^79^,a | b |
| *Enol2* | a | a | a | a | a^91^,b | a^82^,b | a | a |
| *Est* | c^75^,b^18^,a | b^98^,a | b | b | b | b | b | b |
| *Fum* | b | b^99^,d | b^87^,a | b | b^96^,a | b | b^88^,a | b |
| *Gapd* | d | a^82^,b^13^,d | b | d | d^98^,a | d | d^94^,a | d |
| *Gda* | d | c^94^,b | c | c | c | c | - | - |
| *Glo* | b | b^89^,a | a | b | b | b | b | b |
| *Got1* | a | a | a | a | a^87^,b | a | a | b |
| *Got2* | c | c^99^,d | c | c | c^91^,b^8^,d | c^82^,b^16^,d | c | c |
| *Gp* | a | b | b | b | b | b | b | b |
| *Gpi1* | b | b^96^,d^3^,a | b | b | c | c | c | c |
| *Gpi2* | c^96^,a | c^97^,d^2^,e | c | c | c^98^,f | c^95^,f | c | c |
| *Gsr* | d^96^,f | d | d | e | c | c | c | c |
| *Idh1* | c | c^99^,a | c | c | c | c | c | c |
| *Ldh2* | c | c^99^,d | c | c | c^99^,a | c^98^,a | c | c |
| *Mdh2* | b | b | b^87^,a | b | b | b | b | b |
| *Me1* | c^86^,b | b^97^,d^2^,c | d^50^,b^38^,c | b^92^,a | b^97^,a^2^,c | b^93^,a^5^,c | b | b |
| *Me2* | b | b^99^,a | b | b | b | b | b | b |
| *Mpi* | d | d^97^,bcf^1^ | d | b | d^81^,b | d | b^50^,d | d |
| *Ndpk1* | c | b | b | b | b | b | b | b |
| *Ndpk2* | b | b^72^,a | a | b | b^99^,c | b^98^,c | b | b |
| *Np* | b^79^,a^14^,d | b^99^,c | b^50^,a^33^,d | b | b | b | b | b |
| *PepA1* | b | b^99^,c | b | b | b^99^,a | b | b^97^,a | b |
| *PepA2* | b | b^93^,a^4^,d^2^,e | e | b | b^94^,c | b^89^,c | b | b |
| *PepB* | c | c^94^,d^5^,b | a^88^,c | c | c^73^,d^14^,b | c | c^65^,d | b |
| *PepD1* | b | b^99^,a | b^50^,a^38^,d | b^75^,d | b^97^,c | b | b^91^,c | b |
| *PepD2* | b | b^96^,a | b | b | b | b | b | b |
| *6Pgd* | b^96^,c | b | b | b | b^98^,a | b^95^,a | b | b |
| *Pgk* | b^75^,a | b^98^,a | b | b | b | b | b | b |
| *Pgm1* | b | b^98^,c | b | b^94^,a | b^99^,a | b^98^,a | b | b |
| *Pgm2* | b | b^99^,c | c | d^75^,b | b | b | b | b |
| *Pk1* | b | b | b | b | b^99^,c | b^98^,c | b | b |
| *Pk2* | a^96^,b | a | a | a | a | a | a | a |
| *Sod* | a | a | a | a^72^,b | a | a | a | a |
| *Tpi2* | b^75^,c | b^97^,c^2^,a | b | b | b | b | b | b |
| *Ugpp* | a | a | a | a | a^99^,b | a^97^,b | a | a |

Supplementary Table 4. Allozyme frequencies for the two genetic subpopulations identified by Principal Coordinates Analysis within *G. maculatus*, with maximum sample sizes for each (candidate) taxon in brackets. Also shown is a comparative allozyme profile for its sister species *G. rostratus*. Invariant loci: *Ak*, *Ald1*, *Ap*, *Enol1*, *Gapd*, *Got1*, *Idh2*, *Ldh1*, *Mdh1*, *Ndpk1*, *Ndpk2*, *PepB*, *Pgam*, *6Pgd*, *Pk1*, *Pk2*, *Sod*, and *Tpi*. Format as for Table 2.

| Locus | *G. rostratus*  (3) | *G. maculatus*  Lake Hiawatha  (8) | *G. maculatus*  all other sites  (67) |
| --- | --- | --- | --- |
| *Acon* | c^67^,b | c^94^,b | b^70^,c^29^,a |
| *Acp* | a | b | b |
| *Acyc* | - | b^63^,d | b^90^,d^5^,c^4^,a |
| *Ada* | d^83^,c | c^69^,b | c^63^,b^25^, ae^4^,g^2^,df^1^ |
| *Adh* | d | c | c^98^,ab^1^ |
| *Ald2* | b | b^87^,a | b^99^,c |
| *Ca* | b | b | b^97^,a |
| *Ck* | a | a | a^99^,b |
| *Dia* | - | a | a |
| *Enol2* | b | b | b^99^,a |
| *Est* | - | a | a |
| *Fdp* | c^83^,a | b | b^97^,c |
| *Fum* | c | c | c^98^,ab^1^ |
| *G6pd* | b | b | b^89^,a^9^,c |
| *Glo* | a | a | a^99^,b |
| *Got2* | c | b^94^,c | b^57^,c^42^,a |
| *Gp* | a | c | b |
| *Gpi1* | b | b | b^98^,ac^1^ |
| *Gpi2* | a | a | a^93^,b |
| *Gsr* | b | b | b^97^,acd^1^ |
| *Idh1* | b | b^64^,e^29^,d | b^84^,a |
| *Ldh2* | b | b | b^99^,a |
| *Mdh2* | b | a | a |
| *Me* | b | e^88^,f | e^70^,f^17^,c^6^,d^4^,abg^1^ |
| *Mpi* | a^67^,c | c | c^97^,b |
| *PepA1* | a | a | a^99^,b |
| *PepA2* | c | c | c^65^,b^34^,a |
| *PepD* | b^67^,a | b | b^84^,c^15^,d |
| *Pgk* | - | a | a^99^,b |
| *Pgm1* | b | b | b^98^,ac^1^ |
| *Pgm2* | - | b^58^,a | b |
| *Sordh* | b | a | a |
| *Tpi2* | b | c | c^96^,ab^2^ |
| *Ugpp* | b | b^93^,a | b |
